# Treatment of Sprains

**Published:** 1908-05

**Authors:** Theodore Toepel

**Affiliations:** Lecturer on Medical Gymnastics at the Atlanta College of Physicians and Surgeons, Director of Physical education in the Atlanta Public Schools, Medical Examiner at the Atlanta Athletic Club


### TREATMENT OF SPRAINS.*

BY THEODORE TOEPEL, M. D., LECTURER ON MEDICAL GYMNASTICS AT THE ATLANTA COLLEGE OF PHYSICIANS AND SURGEONS, DIRECTOR OF PHYSICAL EDUCATION IN THE ATLANTA PUBLIC SCHOOLS, MEDICAL EXAMINER AT THE ATLANTA ATHLETIC CLUB.

Sprains are the most common form of injury to a joint. A sprain is a wrenching of a joint, producing a stretching or laceration of the ligaments. It is most frequent in the wrist, knee, and ankle joints. It may be slight and the symptoms subside quickly, or it may be severe and of uncertain length of duration.

*Read before the Georgia Medical Association, Fitzgerald, April 15, 16, 17, 1908.

The synovial membrane is compressed on one side, while on the other it is unfolded, stretched or torn. Ligaments are usually stretched on one side only. Some of the fibres are torn, and sometimes the whole ligament may be detached from its osseus attachment, and even small parts of the bone may be torn off.

There is more or less laceration of vessels with attendant hemorrhage into the joint cavity and the surrounding tissues, in consequence of which the limb in a few days becomes discolored for some distance above and below the joint.

The sprain is rapidly followed by swelling and inflammation of the joint and investing tissues, often very chronic and tedious. As the inflammation subsides, stiffness and pain in using the part continue for a considerable length of time, and are in some cases followed by rigidity and wasting of the limb. In individuals of a rheumatic or gouty habit of body, the inflammation of the joint resulting from the sprain is often most tedious and chronic, and will yield only to appropriate constitutional treatment. In strumous subjects, destructive disease of the joint may ultimately be induced.

Upon the receipt of a sprain and if, after a careful examination, you find no fracture, immerse the part in hot water, or have hot compresses applied for one hour, or place the part in a dry hot air apparatus and expose it to the dry hot air, the temperature being about 300-F, for one hour, to relax the tension, then treat with massage, beginning with gentle friction, gradually increasing in force (as a peripheral nerve sedative), follow with gentle kneading, long continued, beginning at a distance from the injury and gradually approaching it. End with palmar percussion. Bandage tightly with wet bandage, then order ice bags to reduce the inflammation, and insist on perfect rest. Keep the joint elevated for twelve to twenty-four hours, in order to limit the formation of passive congestion in or around the joint.

On the second day, repeat the treatment of first day, omit ice and add passive circumduction and passive flexion and extension. On the third day, use treatment of the first two days with resistive movements added. Encourage activity between treatments after the second day. Remove the bandage permanently the third or fourth day. A light sprain takes from seven to ten days to cure, though one of hip and shoulder requires longer time, and it is safer not to use them for one week.

Except in cases of unusually severe sprains, where ligaments are torn or detached, movements of the joint, passive, active and resistive in their order, when properly supported, are very beneficial. When there is much effusion in the joint, the limb should be kept elevated, and active motion of the joint suspended until the effusion has subsided. If the injury has been severe, where tendons have been torn across or have become detached, and the part is very sensitive to motion or jar, the joint having been protected with gauze, is fixed in a light plaster bandage. This is cut after the eighth day, to allow for daily massage and exposure to hot air, which is of great service in hastening the absorption of the effusion. Care must be taken to return the limb to the cast after each treatment in exactly the same position as when the cast was first moulded to the part. In most cases, this procedure must be continued for about four weeks. I consider this method superior to that of applying adhesive strips, where the physician is unable to do anything more for his patient, who in many cases is left to rely upon nature to effect a doubtful cure. The use of massage and hot air is of great service in relieving the discomfort, and especially in stimulating the circulation of the blood, upon which repair depends. As soon as practicable, begin the use of active and resistive exercise to prevent stiffness and to strengthen the weakened tendons and ligaments. Resistive exercises are especially indicated in this condition, because of the necessity of localized movements, which must be confined to the afflicted tendons and ligaments.

A chronic sprain may be the result of an inefficiently treated acute injury, in which an improper attitude, originally assumed to spare the sensitive part, finally becomes habitual. In other instances, persistent disability may be the result of fixation of the joint for too long a time in splints. Such disuse causes atrophy of the muscles and of the bones as well, while the effused material within and without the joint remains, because of the improper circulation. The same disability may follow simple disuse of the injured part. It is more often observed in nervous individuals, who exaggerate the importance of the injury, and the discomfort that it causes. In such cases, the limb may be discolored by venous congestion, the part may be œdematous, and the movements may be limited by adhesions, or by muscular adaptation to the habitual attitude.

Treatment of chronic sprains must be conducted with the aim of restoring the normal range of motion and so supporting the part that normal functional use may be permitted. If adhesions have formed, and if the part is persistently held in an abnormal attitude, forcible manipulation under anæsthesia may be required as a preliminary treatment, followed by fixation for a time in a plaster bandage, in the attitude directly opposed to that which has been habitual. When all discomfort has disappeared, a support should be worn for a time. The most effective after treatment is passive and active exercise.

In conclusion, allow me to say that notwithstanding the fact that the traditions of the profession required absolute rest of the affected parts after injury to the joint, we now know conclusively, that massage and then exercise applied early and with a suitable degree of skill and perseverance, effects a more speedy cure in most cases of sprains than absolute immobility, and prevents both the loss of movement which usually occurs, and the muscular atrophy which is the natural result of absolute rest and immobility.

